# Editorial: Upgrading the classic: the transformation of rudimentary fermented products into controlled industrial processes

**DOI:** 10.3389/fmicb.2023.1291671

**Published:** 2023-09-26

**Authors:** Dão Pedro de Carvalho Neto, Gilberto Vinícius de Melo Pereira, Adewale Olusegun Obadina, Pushpa S. Murthy

**Affiliations:** ^1^Federal Institute of Education, Science, and Technology of Paraná, Londrina, Brazil; ^2^Department of Bioprocess Engineering and Biotechnology, Federal University of Paraná, Curitiba, Brazil; ^3^Department of Food Science and Technology, Federal University of Agriculture, Abeokuta, Nigeria; ^4^CSIR-Central Food Technological Research Institute, Mysore, India

**Keywords:** traditional fermentation, starter cultures, food safety, fermentation optimization, microbial diversity monitoring

Over the course of human evolution, the creation of fermented foods and beverages has been instrumental in molding our culture, society, and even our physiological development. By breaking down carbohydrates into smaller molecules through fermentation, humans have harnessed microorganisms to produce an array of foods such as beer, wine, yogurt, cheese, and various other delicacies. This process not only enhanced the preservation of different substrates but also improved their sensory and nutritional qualities. Thus, fermentation has experienced a surge of innovation in recent times due to advancements in technology and a growing awareness of its potential benefits for food production and sustainability. The transition from fermentative processes conducted in earthenware jars sealed with grass to stainless steel bioreactors boasting meticulous control over aeration, agitation, and temperature marks a technological leap. This advancement has streamlined the standardization and industrial scaling of a variety of fermented foods, such as cheese, beer, bread, wine, and yogurt, collectively contributing to a global market valued over US$333 billion.

Nonetheless, several significant fermented food commodities continue to be produced using traditional methods, including cocoa, coffee, kombucha, and *kimchi*. These products are performed using rudimentary techniques in improvised fermentation vessels or tanks, without control of temperature or the use of starter cultures.

To contribute to the transformation of this scenario, the present Research Topic encompasses recent studies that brings out new insights and strategies that can be applied in the future to aid in the industrialization of traditional fermented food and beverages.

The growing adoption of the vegetarianism and veganism practice has been dictating the food market's search for plant-based products. Within this Research Topic, Ziarno et al. conducted an evaluation of the potential for introducing 16 commercially available lactic acid bacteria strains, typically employed in the dairy industry, into soy beverages, and assessed their influence on various compositional aspects during storage. Soy beverages were fermented during using three different groups: (i) classical yogurt strains (*Streptococcus thermophilus, Lactobacillus delbrueckii* subsp. *bulgaricus*); (ii) classical yogurt strains added with unconventional lactic acid bacteria; (iii) and monocultures. The temporal analysis of acidification revealed that monocultures showed a lower level of acidification rate of monocultures, while groups I and II achieved maximum acidification rate ranged from 0.006 to 0.013 depending on the starter culture tested. Interestingly, during 35 days of storage, the population of all samples achieved the minimum cell count according to FAO/WHO guidelines. Technological aspects of acidity, water holding capacity, texture, and carbohydrate and fatty acids content showed a strain-dependent effect, where YOMIX-207 (*S. thermophilus, L. delbrueckii* subsp. *bulgaricus, L. acidophilus, Bifidobacterium lactis*) and YC-X16 (*Streptococcus thermophilus, Lactobacillus delbrueckii* subsp. *bulgaricus*) showed results comparable to those of traditional yogurt. These findings offer valuable insights that can be leveraged to optimize and better control the production of soy-based yogurt.

During food fermentation, maintaining the presence of higher alcohols at a desired level (<10 g/L) is crucial for their aroma contribution to the final beverage. Nevertheless, when these concentrations exceed the threshold, they can lead to headaches and hangovers. In an effort to advance theoretical knowledge in the field. Wang C. et al. investigated the correlation between composition of glutinous and non-glutinous rice varieties and the formation of n-propanol, isobutanol, isoamyl alcohol, and phenylethnanol. They utilized a low higher alcohol-producing *Saccharomyces cerevisiae* strain for their research. The study revealed that the metabolic peak of the inoculum was at 2 days of fermentation process with high ethanol formation and glucose consumption. Regarding amino acids content, the dynamics was time-dependent, where at the middle point (2 d) a high-consumption rate was observed, while at the end point (17 d) a high hydrolyzation rate was observed, where valine, leucine, phenylalanine, arginine, glycine, and tyrosine concentrations exceeded 400 mg/L in some cultivars. The higher alcohol content was significantly lower in glutinous rice types. A correlation analysis was conducted to assess the influence of the Ehrlich and Harris pathways on the formation of higher alcohols. The analysis revealed significant correlations (>0.6) in only three cases across all 10 rice wine fermentations: valine–isobutanol, glucose–isoamyl alcohol, and glucose–phenylethanol. Significant correlations with other amino acids were observed on a cultivar-dependent effect. The study also showed the significant impact of the Harris pathway in the formation of higher alcohols in rice wine and opens an avenue to investigate the metabolism modulation of *S. cerevisiae* for higher alcohol production.

Wang J. et al. established a reliable method to monitor the population dynamics and quantify the viable cells of *Lactiplantibacillus plantarum* in wine fermentation combining the propidium monoazide (PMA) with CELL-qPCR technique. A concentration of 25 μM of PMA showed better interaction with dead cells in comparison to lower concentrations and did not interfere with the amplification of live cell DNA. The PMA-CELL-qPCR treatment efficiency was not affected when dead-to-live cells ratio was below 10^2^ and the false noise of dead cells from the qPCR was eliminated. The study also revealed that PMA-CELL-qPCR showed no differences in comparison to plate counts, while qPCR without PMA treatment could reach differences of up to 3 log UFC/mL. The study proposes a reliable and accurate method to quantify the specific measurements of viability of starter cultures in targeted fermentations. Further studies should focus on evaluate the stability of the method in different fermentation matrices.

Food production environment is a rich source of nutrients not only for target microorganisms, but also for foodborne pathogens that represents a major threat for consumers and the economy. In order to address this issue, Liu et al. evaluated the effects of *Streptomyces parvus* compounds against *Vibrio parahaemolyticus*, the second most prevalent seafood pathogen. Based on the core pangenome constructed using 58 complete sequences of *V. parahaemolyticus* and a subtractive proteomic analysis against homologous human protein, the authors revealed that the Flagellar motor switch protein (FliN) was a suitable candidate for drug targeting. A *in situ* molecular docking study using an in-house compound library revealed that Actinomycin D exhibited H-bonds and non-bonded interactions between the active site of the Actinomycin D and amino acids residues from the FliN protein. Validation studies confirmed a pronounced inhibitory effect over *V. parahaemolyticus* when exposed to 16 μg/mL of Actinomycin D, besides a complete interruption of swimming and swarming motility at 8 μg/mL. Inhibition of biofilm formation and EPS production were also observed.

In summary, the articles assembled within this Research Topic offer brings out new perspectives of optimization in traditional fermented beverages, methodology for target microorganisms monitoring during fermentation process, and the prospection of drugs against foodborne pathogens. Standards, safety, and regulations of the final product, besides clean labeling, circular bioeconomy, and thus sustainability will be the important imprints of the fermentation industry. Further research is warranted to investigate the influence of indigenous microbiota on the ultimate quality of fermented products and to address safety concerns associated with rudimentary fermentation processes.

## Author contributions

DC: Writing—original draft. GM: Writing—original draft. AO: Writing—review and editing. PM: Writing—review and editing.

